# Approaches Mediating Oxytocin Regulation of the Immune System

**DOI:** 10.3389/fimmu.2016.00693

**Published:** 2017-01-10

**Authors:** Tong Li, Ping Wang, Stephani C. Wang, Yu-Feng Wang

**Affiliations:** ^1^School of Basic Medical Sciences, Harbin Medical University, Harbin, China; ^2^Department of Internal Medicine, Albany Medical Center, Albany, NY, USA

**Keywords:** cytokine, hormone, hypothalamus, immune, oxytocin, thymus

## Abstract

The hypothalamic neuroendocrine system is mainly composed of the neural structures regulating hormone secretion from the pituitary gland and has been considered as the higher regulatory center of the immune system. Recently, the hypothalamo-neurohypophysial system (HNS) emerged as an important component of neuroendocrine–immune network, wherein the oxytocin (OT)-secreting system (OSS) plays an essential role. The OSS, consisting of OT neurons in the supraoptic nucleus, paraventricular nucleus, their several accessory nuclei and associated structures, can integrate neural, endocrine, metabolic, and immune information and plays a pivotal role in the development and functions of the immune system. The OSS can promote the development of thymus and bone marrow, perform immune surveillance, strengthen immune defense, and maintain immune homeostasis. Correspondingly, OT can inhibit inflammation, exert antibiotic-like effect, promote wound healing and regeneration, and suppress stress-associated immune disorders. In this process, the OSS can release OT to act on immune system directly by activating OT receptors or through modulating activities of other hypothalamic–pituitary–immune axes and autonomic nervous system indirectly. However, our understandings of the role of the OSS in neuroendocrine regulation of immune system are largely incomplete, particularly its relationship with other hypothalamic–pituitary–immune axes and the vasopressin-secreting system that coexists with the OSS in the HNS. In addition, it remains unclear about the relationship between the OSS and peripherally produced OT in immune regulation, particularly intrathymic OT that is known to elicit central immunological self-tolerance of T-cells to hypophysial hormones. In this work, we provide a brief review of current knowledge of the features of OSS regulation of the immune system and of potential approaches that mediate OSS coordination of the activities of entire neuroendocrine–immune network.

## Introduction

Immune activities are regulated by many factors, such as the genetic individual variations, immune cytokine, hormone, emotion, nutrition, metabolism, sleep, age, neural activity, and pathogens. Among them, neuroendocrine regulation of immune system is the fundamental machinery (1, 2). Recently, the hypothalamic oxytocin (OT)-secreting system (OSS) has emerged as a pivotal factor in neuroendocrine regulation of immune activities (3). However, its relationship with other hypothalamic–pituitary–immune axes as well as peripherally produced OT remains unclear, which is further explored in this review.

## Neuroendocrine–Immune Network and the OSS

### The Neuroendocrine–Immune Network

As early as 1977, the existence of a neural–endocrine–immune network has been proposed (4). In this network, immune activity can influence the development (5) and functions (6) of rat hypothalamus, the higher control center of the neuroendocrine system. Conversely, changes in neuroendocrine activities can affect the immune response through pituitary tropic hormones and the autonomic nervous system (7). This bidirectional communication between hypothalamic neuroendocrine system and the immune system forms a neuroendocrine–immune network.

### The OSS–Immune Network

In the neuroendocrine–immune network, immune regulatory roles of the hypothalamo-neurohypophysial system (HNS) (8), particularly its OSS, have been considered critical (3). The OSS is mainly composed of magnocellular OT neurons in the supraoptic nucleus (SON), paraventricular nucleus (PVN), and several accessory nuclei of the hypothalamus as well as their axon terminals in the posterior lobe of the pituitary. In addition, parvocellular OT neurons in the PVN, a major source of OT in the brain and spinal cord, coexist with corticotropin-releasing hormone (CRH) and thyrotropin-releasing hormone (TRH) neurons in the PVN while closely interacting with magnocellular OT neurons (9) and the autonomic center that can regulate immune activity through sympathetic nervous system (10). In this OSS–immune network, the magnocellular OT neurons in the SON play a dominant role in response to immune challenges as shown in rat sepsis (11).

## Characteristics of the OSS–Immune Network

### The OSS Is Involved in the Development and Functions of the Central Immune Organs

It has been reported that neurointermediate pituitary lobectomy, blocking the secretion of neurohypophysial hormones including OT, significantly changed humoral and cellular immune responses in rats (12, 13). OT can also promote the formation of human hematopoietic stem cells (14) and promote rat bone marrow mesenchymal stem cell migration (15). Moreover, blocking OT receptor (OTR) signaling can inhibit the differentiation of mouse thymic T-cells (16) and estrogen-evoked bone formation (17) while increasing the expression and secretion of inflammatory cytokines, such as interleukin (IL)-6 in human amnion (18). Thus, OT is a key regulator of the immune system and thus can extensively regulate immune activity (3), which is considered to be mediated by OTRs as summarized in Table 1.

**Table 1:** **Major immune functions of the oxytocin-secreting system (OSS)**.

Sources	Targets	Effects	Reference
**Development of the immune system**			

Human, mouse	Osteoblast	Bone mass ↑	(17)

Rat	BMSC	Intracellular [Ca^2+^] ↑	(19)

Rat	MSC	Apoptosis ↓	(20)

Human, rat, and mouse	Thymus	Clone deletion of self-reactive T-cells ↑	(1, 16)

Mouse fetus	Thymic organ cultures	Survival of thymic CDS cells ↑	(21, 22)

Rat	UCB-MSC	Migration of BMSC to the injured area ↑	(15, 23)

Parturient women	Blood	Number of B-lymphocyte ↑	(24)

**Immune surveillance**			

Rat at early stage of sepsis	Brain, plasma	OT levels ↑, OT in the SON and neurohypophysis ↓	(25)

Rats of acute pancreatitis	Brain	Brain OT release ↑	(26)

Rats with advanced cancer	The OSS	Fos expression in OT neurons ↑	(27)

Rats with adjuvant arthritis	SON, PVN	OT mRNA ↑	(28)

Human lung and GI tumors	Lung, liver	OTR in tumor tissues ↑	(29, 30)

Breast cancer	OT levels	Pituitary and blood ↑; cancer tissues ↓	(31)

**Immune defense**			

Humans and animals	Immune cells, blood	Inflammatory cytokines, e.g., nitrite, TNF-α, and IL-1β levels ↓; oxidative stress ↓; apoptotic pathways ↓; immune damages, activation of free radical damaging cascades and lactate dehydrogenase ↓; excessive infiltration of neutrophils ↓	(25, 32–36)

Human	Plasma	ACTH, cortisol, procalcitonin, IL-1, IL-4, IL-6, macrophage inflammatory protein-lα and 1β, monocyte chemoattractant protein-1, interferon-inducible protein 10, and vascular endothelial growth factor ↓	(37)

Human	Skin	Antibacterial effect of antibiotics ↑	(38)

Human	Skin	Wound healing ↑	(39)

Rat	Stomach	Antisecretory and antiulcer effects ↑	(40, 41)

Rat	Peripheral neuron	Harmful effects of hyperglycemia ↓	(32)

Mice	CD157 signaling	Mental disorders associated with immune disorders ↓	(42)

**Immune homeostasis**			

Rat	DM-MSC	Angiogenic capacity	(43)

Rabbit	Myocardial cell	Antifibrotic and angiogenic effect	(44)

Rat and swine	Brain	Autoantibodies in multiple sclerosis are reactive with OT neurons	(45)

Diabetic rats	Muscle, pancreas	Regenerative capacity of skeletal muscle and pancreatic islet cells ↑	(46, 47)

Caco2BB gut cells	Enterocyte	Inflammation-evoked apoptosis ↓	(48)

HIV-infected patients	Blood	CD4^+^ cell counts ↑	(49)

*ACTH, adrenocorticotropic hormone; BMSC, bone marrow stromal cells; DM-MSC, diabetic bone MSC; MSC, bone marrow mesenchymal stem cell; IL, interleukin; OT, oxytocin; OTR, OT receptor; PVN, paraventricular nucleus; SON, supraoptic nucleus; TNF-α, tumor necrosis factor-alpha; UCB-MSC, umbilical cord blood-derived mesenchymal stem cell*.

### The OSS Bidirectionally Communicates with the Immune System through Multiple Approaches

**Figure 1:**
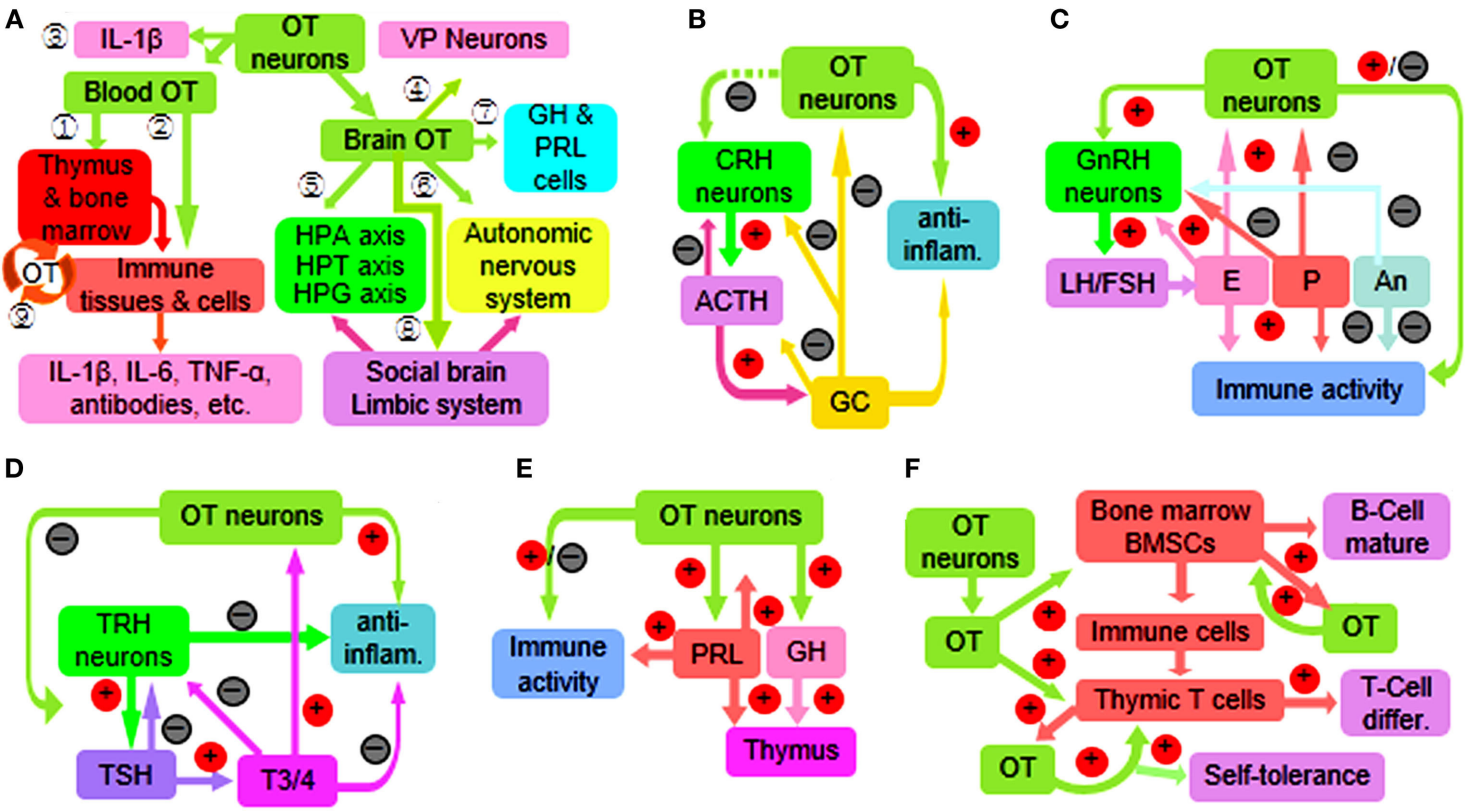
**Diagram of immune functions of the oxytocin-secreting system (OSS) through a variety of approaches**. **(A)** Overview of the approaches. The circled numbers 1–9 represent the effects of OT on the immune system through activating peripheral OTRs on central immune organs (1) and peripheral immune organs, tissues, and cells (2); and secretion of IL-1β (3) as well as *via* centrally acting on VP neurons (4); the hypothalamic–pituitary–adrenal (HPA) axis, hypothalamic–pituitary–thyroid (HPT) axis, and the hypothalamic–pituitary–gonadal (HPG) axis (5); autonomic nervous system (6); growth hormone (GH) and prolactin (PRL) (7); and social brain and the limbic system (8). In addition, peripherally produced OT also exerts some autoregulatory effects (9). **(B)** Interactions between the OSS and HPA axis. Note that plus sign in red circle and minus sign in black circle represent facilitation and inhibition, respectively; the dashed line in green indicates multiple approaches. **(C)** Interactions between the OSS and HPG axis. **(D)** Interactions between the OSS and HPT axis. **(E)** Interactions of the OSS with GH and PRL. **(F)** Synergic effects of the OSS and peripherally produced OT on the immune system. Abbreviations: ACTH, adrenocorticotropic hormone; An, androgens; differ., differentiation; inflam., inflammation; E, estrogens; FSH, follicle-stimulating hormone; GC, glucocorticoids; IL, interleukin; LH, luteinizing hormone; P, progesterone; T3/4, triiodothyronine and thyroxine; TNF-α, tumor necrosis factor-α; TSH, thyroid-stimulating hormone; VP, vasopressin.

Oxytocin can regulate immune functions (37) by activating OTRs directly (3) and through sympathetic outflow (10, 50) that is known to control the activity of rat thymus (51) and bone marrow (52). OT can also change the activity of other hypothalamic–pituitary–immune axes (Figure 1A). Conversely, the OSS is also the target of immune diseases. For example, OT neurophysin shares an antigen with human lung carcinoma LX-1 cells (53); OT neurons are a major target of many autoimmune diseases such as multiple sclerosis (45, 54); OT in hypothalamic neurons decreased in HIV-infected patients (55). In response to immune challenges, IL-6 (50) and IL-1β (56) can activate rodent OT neurons in the PVN and/or SON, while microglia in the PVN can increase OT secretion and sympathetic activity (57). Thus, the OSS can regulate immune activity more accurately.

### OT Neurons Are “Immune Cells” and Mainly Function through OTRs

Oxytocin neurons can produce cytokines such as IL-1β (58), nitric oxide (59), and prostaglandins (60, 61) in rats. These cytokines can not only autoregulate OT neuronal activity, such as nitric oxide (62) and prostaglandins (61) in rats, but also extensively modulate immune activity of other brain structures (63) (Figure 1A).

Both the OSS and the immune system can synthesize and release neurotransmitters, neuropeptides, and cytokines while expressing receptors for both neuropeptides and immune cytokines including OT and OTRs (1, 2). OTRs are widely identified in immune organs, tissues, and cells, such as rat thymic epithelial cells (64) and bone marrow stem cells (19). Importantly, the expression of OTRs in immune tissues can be inducible, which has been shown in bovine peripheral blood mononuclear cells and T lymphocytes (65), rat mesenchymal stem cells (19), and gut (48). Thus, OT can modulate immune activity and immune-regulating cells directly and dynamically to meet the demands of a variety of immune challenges.

### The OSS Behaves As an Integrative Organ in Feedforward and Feedback Immune Loops

Oxytocin neurons can integrate information from presynaptic neurons, detect the state of astrocytic plasticity and microglial activation, sense concentrations of blood-borne substances and local neurochemical including cytokines (3, 66–68), and in turn secrete appropriate amount of OT into the blood and brain. This could preset the immune system in an optimal working condition through regulating the activity of bone marrow, thymus, and T-/B-cells as well as other immune organs and tissues (3). In parallel, overly increased immune challenges can be suppressed through increasing OT release. For example, IL-1β released by immune cells can activate OT neurons or promote the release of OT into the blood in rats (69, 70); OT subsequently reduces the production of inflammatory cytokines as evidenced in men (37), thereby maintaining the homeostasis of immune functions and inhibiting immune damages.

## Immune Functions of the OSS

The OSS is involved in many physiological and pathological immune processes (Table 1), which falls into the following categories.

### Immune Surveillance

The OSS can detect immune states and serves as biomarker of immune challenges. For instance, it has been identified in rats that there is significant increase in plasma OT levels at the early stage of sepsis (25), brain OT release following pancreatic injury (26), OT mRNA in adjuvant arthritis (28), and Fos expression in the OSS in advanced cancer (27). Thus, increased OT levels manifest immune disturbance.

### Strengthening Immune Defense

Body’s immune defense is carried out through multiple levels of immune machineries. OT can strengthen the physical and chemical barriers through suppressing proinflammatory cytokines (34) and promoting wound healing (39) in human skin, enforce human non-specific cellular and humoral immunity *via* strengthening the antibacterial effect of antibiotics (38) and accelerating migration of rat bone marrow mesenchymal stem cells to the injured area (15), and increase acquired immunity by promoting the differentiation of mouse thymic cells (16). OT was also found to alleviate harmful effects of hyperglycemia on rat peripheral neurons by suppressing inflammation, oxidative stress, and apoptotic pathways (32). As a result, activated OSS can adjust inflammatory reactions at appropriate levels to prevent body from immune damages.

### Maintenance of Immune Homeostasis

A healthy individual may fall into diseases due to excessive or insufficient immune activity. Theoretically, the regulatory effects of OT on immune responses should allow OT to influence the progress of autoimmune diseases, which is supported by the finding that in women living with HIV, high levels of OT were positively associated with CD4^+^ cell counts (49). Moreover, OT was found to increase the production of hematopoietic stem cells and the survival of thymic CD8 cells (22) while reducing the infiltration of neutrophils in rats (33, 36) and the production of human inflammatory cytokines (34). Thus, OT is critical in maintaining immune homeostasis.

### Other Immune Functions

The OSS can also influence other immune processes. For example, OT can improve autism, depression, and other mental disorders associated with immune disorders (71) and increase resistance of enterocyte apoptosis (48) while reducing the apoptosis of rat mesenchymal stem cells (20), and promoting regenerative capacity of skeletal muscle (46) and pancreatic islet cells of diabetic rats (47).

### Adverse Effect

It is worth noting that OT can worsen immune injury at parturient women with latex allergy and bronchial asthma (72), chorioamnionitis (73), and premature birth (74). This is likely associated with the muscle contraction following OTR activation in these tissues (18, 75) and requires special attention to the application of OT in parturient women with related disease histories.

## Relationship Between the OSS and Other Neuroendocrine Regulatory Systems

The neuroendocrine regulation of immune activities has been considered as a function of several hypothalamic neuroendocrine axes, particularly the hypothalamic–pituitary–adrenal (HPA) axis, hypothalamic–pituitary–thyroid (HPT) axis, and the hypothalamic–pituitary–gonadal (HPG) axis. Changes in their activity can change the secretion of glucocorticoids (GC), thyroid hormone, sex steroid hormone, growth hormone (GH) prolactin (PRL), and vasopressin (VP) and thus profoundly affect lymphocyte homeostasis, self-tolerance, and immune pathological processes (76, 77). Importantly, there are close associations between activities of the OSS and these axes in the neuroendocrine regulation of the immune system. Additionally, the contribution of peripherally produced OT, particularly intrathymic OT, to the OT-associated immune activity should also influence the immune functions of the OSS.

### The OSS and Hypothalamic–Adenohypophysial–Immune Axes in Immune Regulation

The immune regulatory roles of the adenohypophysial hormones (2, 63) are different from the neurohypophysial hormones as indicated by the effects of different types of pituitary lobectomy in rodents on antibody-mediated antimicrobial effects (78) and on antibody- and cell-mediated antiparasite effects (13, 79). Moreover, the OSS has close interactions with the HPA, HPT, and HPG axes (Figure 1A).

#### The OSS and HPA Axis

The immune function of HPA axis is mainly at suppression of immune reactions by offsetting the inflammatory reaction while activating anti-inflammatory processes (80–82). Experiments in rats further revealed that GC can rapidly inhibit the hypothalamic neuroendocrine activities including the secretion of CRH and OT (83). By contrast, OT can inhibit the activation of HPA axis induced by some stress stimuli (84) and their associated maternal depression (85) in rats. This is consistent with the finding that maternal separation decreased rat OSS activity (85, 86) while increasing the activity of HPA axis in calves (87). However, the OSS and HPA axis could work synergistically through suppression of inflammatory reactions by corticosteroids and OT, respectively (Figure 1B).

#### The OSS and HPT Axis

Thyrotropin-releasing hormone can directly regulate the immune activity as seen in mouse allergic encephalomyelitis (88) and in patients with Hashimoto’s thyroiditis and primary hypothyroidism (89). It is also reported that triiodothyronine plays a critical role in controlling the maturation and antitumor functions of mouse dendritic cells and stimulation of cytotoxic T-cell responses (90). There is also evidence showing a close interaction between the OSS and the HPT axis. For example, high dose of triiodothyronine can increase OT mRNA levels in rat PVN (91) and OT release from rat pituitary (92). On the contrary, OT can reduce the response of pituitary thyroid-stimulating hormone cells to TRH and then reduce the release of thyroid hormone in rats (31) (Figure 1C).

#### The OSS and HPG Axis

The HPG axis is mainly involved in immune responses during sexual activity, menstrual cycle, and pregnancy (93). Estrogen can activate the immune response and even cause autoimmune diseases, such as lupus erythematosus, while androgen plays a role in human immune suppression (94). On the one hand, OT can stimulate the secretion of gonadotropin-releasing hormone directly by activating rat gonadotropin-releasing hormone neurons (95). On the other hand, the OSS is modulated by sex steroid hormones. For example, allopregnanolone suppresses (56) and estrogen increases (96) the activity of magnocellular OT neurons and/or OT secretion (Figure 1D). Noteworthy is that the interactions between the OSS and HPG axis could vary in females at reproductive age due to the variations of hormonal interactions at different stages of reproduction (70, 97).

### Comparison of Immune Regulatory Effects of VP versus OT

The VP-secreting system (VSS) and OSS coexist in the HNS (68), and thus, the VSS could also be involved in the immune effects of rat neurointermediate lobectomy (12, 13). In fact, the VSS does have certain immune functions that are often opposite to the OSS (68). For example, in rat tissue culture, VP inhibits and OT facilitates the growth of thymus gland (98). Moreover, the immune functions of the VSS are narrower than that of the OSS. For example, the distribution of OTRs in the immune system is more extensive than that of VP receptors as seen in rats (99) and in mice (21). In contrast to the extensive immune functions of the OSS (Table 1), blocking VP signaling can only block the production of interferon-γ by mouse spleen lymphocytes specifically and reversibly (100) along with a few of other functions (68).

Noteworthy are the following exceptions. (1) The VSS can also inhibit immune reaction at brain levels (101) and that is likely due to VP-evoked activation of the HPA axis (82). (2) The OSS and VSS may promote the maturation of immune system sequentially. That is, OT promotes T-cell differentiation in the thymus (16), and VP further facilitates their maturation in the spleen (100). Finally, OT can increase the activity of VP neurons (60), and thus, the functions of VSS can be considered as a supplement to the OSS in immune regulation.

### Relationship between the OSS and Other Neuroendocrine Activities

In addition to the three major hypothalamic neuroendocrine axes and the VSS, other hypophysial hormones, such as GH and PRL, are also involved in neuroendocrine regulation of immune responses (Figure 1E). GH and PRL can improve the proliferation and transplantation of the thymic cells and exert immune promoting effects (102). These two hormones also have close interaction with the OSS. It has been reported that application of OT in rat cerebral ventricles promotes the secretion of GH (103); OT can act on rat adenohypophysis to increase the secretion of PRL that reversely promotes the production of OT (103). This immune regulatory effect of OT *via* GH and PRL is consistent with the suppressive effect of neurointermediate lobectomy on rat thymus development (12, 13) and supports that OT is an essential hormone in the development and functions of the immune system.

### Intrathymic OT versus the OSS in Immune Regulation

Both OT and OTR are expressed in mouse bone marrow (17) and in the thymus (104, 105) as well as many other components of the immune system (106, 107). Thus, peripheral OT has also some important immune functions (Figure 1F). For example, the intrathymic OT can dually regulate T cell-negative and -positive selections (108). Thymic epithelium can present OT and elicit clone deletion of self-reactive T-cells (1), thereby eliciting central immune self-tolerance of T-cells to OT and other hypophysial hormones (108). This function, as well as OT effects on rat bone marrow development (19), indicates that locally produced OT has important role in the maturation of immune system. However, as the thymus involutes over time, the immune functions of local OT mainly serve as a supplemental factor to OSS regulation of the immune system at local levels (77) through hidden secretion (108) or autocrine/paracrine effects (17).

## Conclusion

The OSS plays a key role in the neuroendocrine–immune network. It not only has direct regulatory effects on the development and functions of the immune system but also exerts functions of immune defense and homeostasis through coordinating the activity of the whole neuroendocrine–immune network as well as peripherally produced OT (Figure 1). The main question remaining to be answered is still the details of its relationship with other components in the neuroendocrine–immune network and peripherally produced OT under different types and extents of immune challenges. Answering these questions has great theoretical significance and broad potential for medical translation.

## Author Contributions

TL and PW wrote the first draft; SW participated in the revision; and YFW designed the review and made the final revision.

## Conflict of Interest Statement

The authors declare that the research was conducted in the absence of any commercial or financial relationships that could be construed as a potential conflict of interest.
